# Prevalence of cardiovascular risk factors among adults without obvious cardiovascular disease in a rural community in Ekiti State, Southwest Nigeria

**DOI:** 10.1186/1471-2261-13-89

**Published:** 2013-10-20

**Authors:** Olarinde J Ogunmola, Adeleke O Olaifa, Olutoyin O Oladapo, Oluwole A Babatunde

**Affiliations:** 1 Department of Internal Medicine, Cardiac Centre, Federal Medical Centre, Ido-Ekiti, Ekiti State, Nigeria; 2 Department of Internal Medicine, Federal Medical Centre, Ido-Ekiti, Ekiti State, Nigeria; 3 Division of Cardiovascular Medicine, College of Medicine, University of Ibadan, Ibadan, Nigeria; 4 Department of Community Medicine, Federal Medical Centre, Ido-Ekiti, Ekiti State, Nigeria

**Keywords:** Hypertension, Cardiovascular disease, Risk factors, Rural communities, Nigeria

## Abstract

**Background:**

Cardiovascular disease worldwide is largely driven by modifiable risk factors. This study sought to identify and determine the prevalence of traditional cardiovascular risk factors according to sex in inhabitants of a rural community in a developing country.

**Methods:**

This cross-sectional study included participants aged ≥40 years in the rural community of Aaye Ekiti, Ekiti State, Southwest Nigeria. All participants who met the inclusion criteria were drawn from the 161 households in the community. Data on the following were collected: arterial hypertension, diabetes mellitus, obesity, dyslipidaemia, smoking, physical activity, alcohol consumption, and sociodemographic parameters. These were analysed with SPSS version 16.0 software.

**Results:**

The 104 participants (33 male, 71 female) had a mean age (± standard deviation) of 66.77 ± 12.06 years (range, 40–88 years). The majority of the participants (56.7%) were aged 60–79 years. Hypertension was present in 66.4%, diabetes mellitus in 4.8%, abdominal obesity in 38.46%, smoking in 2.9%, physical inactivity in 29.8%, and high alcohol consumption in 1%. Dyslipidaemia, as represented by low HDL-C, occurred in 30%. There were borderline high levels of TC in 4.5%, LDL-C in 1.1%, and TG in 12.5%, but no subject had a high level. Abdominal obesity, alcohol consumption and smoking were statistically significantly associated with sex.

**Conclusion:**

In this study, traditional cardiovascular risk factors, apart from hypertension, obesity, physical inactivity and low HDL-C had a low prevalence in the rural Nigerian community. However, the high prevalence of hypertension in this poor community suggests a high risk of a future cardiovascular event.

## Background

Cardiovascular disease (CVD) is the leading cause of death in adults worldwide [1]. In developing countries, the proportion of worldwide deaths associated with CVD is projected to rise from 28.9% in 1990 to 36.3% by the year 2020 [2]. CVD now causes most deaths in all developing regions, and is the leading cause of death in those older than 45 years in sub-Saharan Africa [3].

CVD worldwide is largely driven by modifiable risk factors. These risk factors include smoking, lack of physical activity, low fruit and vegetable intake, high fat and salt intake, hypertension, abdominal obesity, dyslipidaemia, and excess alcohol intake [3]. The upward trend in CVD in sub-Saharan Africa is likely as a result of the increasing prevalence of some of these modifiable risk factors [3].

The continuing enormous burden of CVD in developed countries, the concerning trends in cardiovascular risk profiles of adolescents and adults, and the emerging increases in CVD in developing countries underscore the crucial need to redouble treatment and prevention efforts [4]. This is particularly important in Nigeria (and by extension, Africa), where the health care expenditure per capita is 23 dollars (4.6% of total Gross Domestic Product) [5]. Therefore, this study sought to identify and determine the prevalence of traditional (conventional) cardiovascular risk factors according to sex and sociodemographic factors, in residents of a typical Nigerian rural community.

To the best of the authors’ knowledge, no known study of this nature has been conducted in Ekiti State, and no recent study has been conducted in Southwest Nigeria to comprehensively assess CVD risk factors. We hypothesised that traditional cardiovascular risk factors were of low frequency in rural dwellers of Ekiti in Ekiti State, Nigeria. This study was conducted to provide epidemiological information on conventional cardiovascular risk factors among rural dwellers, and therefore a useful reference for preventive strategies, policy formulation and further studies.

## Methods

### Study population

This study was a descriptive, cross-sectional survey conducted in Aaye Ekiti, a typical rural community in the Ido/Osi Local Government Area of Ekiti State, Southwest Nigeria. It is situated about 30 km from Ado-Ekiti, the state capital. The major occupation is farming, mainly cultivation of root crops. The community has an estimated population of 1,610, and is divided into four quarters, namely Onala (51 houses), Oke-Ode (28 houses), Odo-ode (22 houses), and Temidire (60 houses), with a total number of 161 houses, which were visited during the enrolment process. Eligible households were defined as one having participants that met the inclusion criteria. A total of 120 participants were enrolled.

The inclusion criteria were: age ≥40 years; no apparent cardiovascular disease; had lived for at least 3 years in the community; and provided consent to participate in the study. The exclusion criteria were: pregnancy and incapacity to give informed consent.

The community consisted mainly of children and elderly inhabitants, but few young adults, the majority of whom had migrated to the cities for white-collar jobs. Those in the community who met the inclusion criteria were encouraged to participate by community leaders, chiefs and announcements in strategic places, e.g., the church, meetings and community gatherings. The only comprehensive health centre available in the community was used to evaluate all subjects recruited for this study.

### Ethical consideration

The project was approved by the committee on ethics of the Federal Medical Centre Ido-Ekiti, Ekiti State. Permission to use the health centre was obtained from the officer in charge. Individual participant written consent was obtained after a thorough explanation was given and understanding was established. Confidentiality was assured to all participants and data used for this study were stripped of personally identifiable information. Treatment (both preventive and curative) was given to all those who were identified as requiring it, and some were referred to appropriate health institutions for further treatment and follow-up.

The following definitions were adopted for this study. Hypertension: persistently elevated blood pressure (BP) ≥140/90 mmHg, based on at least two readings on separate occasions after the initial screening [6-9]. The choice of 140/90 mmHg as a cut-off point is based on the Seventh Report of the Joint National Committee on prevention, detection, evaluation and treatment of high blood pressure (JNC 7) criteria [7]. Diabetes mellitus and impaired fasting blood glucose: diagnosed according to the World Health Organization diagnostic criteria [10]. Rural community: an area where people work or live on a farm or the number of residents is less than 2000 [11]. Dyslipidaemia: National Cholesterol Education Programme Adult Treatment Panel III (NCEP ATP III) cut-off points were used to identify participants with desirable, borderline high and high levels of lipoprotein risk factors [12]—total cholesterol (TC): ≤4.9 mmmol/L, 5–5.9 mmol/L and ≥6.0 mmol/L, respectively; low density lipoprotein cholesterol (LDL-C): <3.24 mmol/L, 3.25–3.98 mmol/L, and ≥4.0 mmol/L, respectively; triglyceride (TG): <1.70 mmol/L, 1.70–2.25 mmol/L, and ≥2.26 mmol/L, respectively; high density lipoprotein cholesterol (HDL-C) cut-off points used to identify participants with protective, borderline, and low levels were: ≥1.5 mmol/L, 1.0–1.4 mmol/L, and ≤0.9 mmol/L, respectively. Classification of body mass index (BMI) [13,14]: normal = 18.5–24.9 kg/m^2^; overweight = 25.0–29.9 kg/m^2^; obese = ≥30 kg/m^2^. Abdominal obesity: waist circumference >102 cm in males or >88 cm in females [14]. Alcohol intake: calculated as the percentage of alcohol by volume multiplied by the volume divided by 1000; low-risk alcohol consumption was defined as a maximum of 3 units per day in females, and a maximum of 4 units per day in males, with at least 2 days per week free of alcohol consumption [15]; higher consumption was considered high risk. Smoking: considered present if smoking was reported up to the day of the interview. Level of physical activity: determined using the modified Hipny Physical Activity Questionnaire (based on the Health Insurance Plan (HIP) of New York questionnaire) [16]; insufficient physical activity was considered if occupation-related activity was less than 15 units on the validated HIP scale, or leisure-related physical activity was less than 4 units on the scale.

### Data collection

All health professionals involved in this study received training in the protocols and in the measurement of all variables involved in this study, to ensure uniformity and minimize errors. A pre-test was undertaken and necessary modification adopted, before the main data collection was performed. Each participant was comfortably seated and assessed for all the listed conventional cardiovascular risk factors as well as taken through a well-structured questionnaire that consisted of sociodemographic characteristics. Height (m) and weight (kg) were measured by standardized techniques and equipment [17]. The reference point used to measure waist circumference with a non-stretchable tape was the highest point of the iliac crest. The narrowest region (visible waist) of the abdomen was used when this region did not coincide with the highest point of the iliac crest [18].

Socioeconomic status was assessed using a general model [19,20] as a convenient index for classification into lower class, middle class and upper class, since the majority of the population were peasant farmers. BP was measured with a mercury sphygmomanometer at least twice in each participant with at least 5 min of rest in between, with the subject seated in a chair and relaxed, the back supported, and the arm at heart level. During the initial screening, BP both arms was measured and the arm with higher BP used subsequently. Tobacco, alcohol and caffeine were not allowed for at least 30 min before taking the measurements.

Fasting blood glucose and fasting lipid profile were determined using capillary blood obtained from a pin prick on finger Pulp using Cardio Check P.A Polymer Technology systems, Inc., a point of care analyser that is a reliable alternative to conventional laboratory devices. Accuracy of the equipment was ±6% compared with standard laboratory equipment.

### Statistical analysis

Data were entered into a statistical computer software package (SPSS version 16.0, SPSS Inc., Chicago, IL, USA). The Student *t*-test was used to determine means ± standard deviation, and the Chi-square test was used for proportions. The Yates correction and Fisher extract test were used when the frequencies expected were lower than five in one or more cells, respectively. In those tests, a p-value of < 0.05 was considered statistically significant.

## Results

In this study, 104 participants were included out of 120 individuals that met the inclusion criteria. Fourteen lacking a fasting lipid profile and two with no random blood sugar results were excluded from further analysis. The final sample consisted of 33 males (31.7%) and 71 females (68.3%), with mean age 66.77 ± 12.06 years (range, 40–88 years). The mean age for males was 69.45 ± 10.36 years (range, 40–85 years), and for females was 65.52 ± 12.64 years (range, 50–88 years). The difference in the mean ages of males and females was not statistically significant (t = 1.559, p = 0.122). None of the menopausal women received hormone replacement therapy. Table 1 presents the demographic characteristics: 26.0% were aged 40–59 years, 56.7% were 60–79 years, and 17.3% were ≥80 years. Most participants were low-income earners and almost all belonged to the lower socioeconomic status. Over half of the participants had no formal education. Nearly all the participants were married and no males were widowed. There were no statistically significant differences in variables relating to sex except for marital status and occupation of participants.

**Table 1:** Sociodemographic characteristics of the participants by sex

**Variables**	**Male (N = 33)**	**Female (N = 71)**	**Total (N = 104)**	**χ**^**2**^	**p-value**
**Age group (years)**					
40–59	7 (21.2)	20 (28.2)	27 (26.0)	0.956	0.62
60–79	21 (63.6)	38 (53.5)	59 (56.7)		
≥80	5 (15.2)	13 (18.3)	18 (17.3)		
**Socioeconomic status**					
Lower class	30 (90.9)	66 (93)	96 (92.3)	2.194	0.33
Middle class	2 (6.1)	5 (7)	7 (6.7)		
Upper Class	1 (3)	0 (0)	1 (1)		
**Highest educational level attained**					
No formal education	14 (42.4)	43 (60.6)	57 (54.8)	5.262	0.15
Primary school	7 (21.2)	16 (22.5)	23 (22.1)		
Secondary school	5 (15.2)	6 (8.5)	11 (10.6)		
Post-secondary school	7 (21.2)	6 (8.5)	13 (12.5)		
**Marital status**					
Single	0 (0.0)	0 (0.0)	0 (0.0)	26.690	0.000
Married	32 (97.0)	33 (46.5)	65 (62.5)		
Divorced	1 (3.0)	1 (1.4)	2 (1.9)		
Widowed	0 (0.0)	37 (52.9)	37 (35.9)		
**Occupation**					
Farming	27 (81.8)	23 (32.4)	50 (48.1)	23.571	0.000
Trading	1 (3)	27 (38.0)	28 (26.9)		
Others	5 (15.2)	21 (29.6)	26 (25.0)		

Data of the traditional cardiovascular risk factors are presented in Table 2. BP measurements showed that 66.4% had systemic arterial hypertension. Systolic BP of all the participants ranged between 100 and 220 mmHg (mean, 150.3 ± 27.9 mmHg). In males, systolic BP ranged between 110 and 200 mmHg (mean, 151.8 ± 22.6 mmHg). In females, systolic BP ranged between 100 and 220 mmHg (mean, 151.80 ± 30.1 mmHg). The diastolic BP of all participants ranged between 60 and 120 mmHg (mean, 87.5 ± 13.7 mmHg). In males, diastolic BP ranged between 60 and 110 mmHg (mean, 87.9 ± 11.6 mmHg). In females, diastolic BP ranged between 60 and 120 mmHg (mean, 87.3 ± 14.7 mmHg). There were no significant differences between the sexes.

**Table 2:** Distribution of traditional cardiovascular risk factors according to sex

**Variables**	**Male (N = 33)**	**Female (N = 71)**	**Total (N = 104)**	**X**^**2**^	**p-value**
	**N (%)**	**N (%)**	**N (%)**		
**BP Category (mmHg)**					
≥140/90	13 (39.4)	31 (28.1)	44 (42.3)	2.095	0.55
<140/90	12 (36.4)	23 (53.5)	35 (33.7)		
≥140/<90	6 (18.2)	16 (18.3)	22 (21.2)		
<140/≥90	2 (6.1)	1 (1.4)	3 (2.9)		
**BMI (kg/m**^**2**^**)**					
<18.5	2 (6.1)	4 (5.6)	6 (5.8)	3.070	0.38
18.5–24.9	17 (51.5)	35 (49.3)	52 (50.0)		
25–29.9	12 (36.4)	19 (26.8)	31 (29.8)		
≥30	2 (6.0)	13 (18.3)	15 (14.4)		
**Tobacco use**					
Non smoker	19 (57.6)	69 (97.2)	88 (84.6)	32.15	0.000
Ex-smoker	13 (39.4)	00 (00.0)	13 (12.5)		
Current smoker	1 (3.0)	2 (2.8)	3 (2.9)		
**Alcohol consumption**					
None	9 (27.3)	63 (88.7)	72 (69.2)	40.247	0.000
Low risk	23 (69.6)	8 (11.3)	31 (29.8)		
High risk	1 (3.0)	0 (0.0)	1 (1.0)		
**Blood sugar category**					
Diabetic	0 (0.0)	5 (7.0)	5 (4.8)	6.723	0.08
Impaired fasting glucose	1 (3.0)	3 (4.2)	4 (3.8)		
Non diabetic	30 (90.9)	63 (88.7)	93(89.4)		
No blood glucose result	2 (6.1)	0 (0.0)	2 (1.9)		
**Waist circumference (cm)**					
≤102 (male), ≤88 (female)	3 (90.9)	34 (47.9)	64 (61.5)	17.617	0.000
>102 (male), >88 (female)	3 (9.1)	37 (52.1)	40 (38.5)		
**Physical activity**					
Active	25 (75.8)	48 (67.6)	73 (70.2)	0.716	0.60
Inactive	8 (24.2)	23 (32.4)	31 (29.8)		

*BP* Blood pressure, *BMI* Body mass index.

Blood glucose values of all participants ranged between 3.4 and 13.6 mmol/L (mean, 4.6 ± 1.5 mmol/L). In males, blood sugar ranged between 3.4 and 5.6 mmol/L (mean, 4.3 ± 0.5 mmol/L), and in females blood glucose ranged between 3.4 and 13.6 mmol/L (mean, 4.8 ± 1.7 mmol/L). In total, five subjects (4.8%) were diabetic, and all were female (Table 2).

BMI ranged between 15.4 and 44.8 kg/m^2^ (mean, 25.0 ± 5.4 kg/m^2^). In males, BMI ranged between 17.0 and 35.0 kg/m^2^ (mean, 24.3 ± 4.3 kg/m^2^), and in females, BMI ranged between 15.3 and 44.8 kg/m^2^ (mean, 25.4 ± 5.8 kg/m^2^). In total, 14.4% were obese, and 29.8% were overweight. There were no significant differences between the sexes.

The proportion of current smokers as shown in Table 2 was low. The proportion of participants consuming alcohol was 30.8%, with only one subject at high risk of alcohol-related diseases. The level of physical activity showed that most of the population were physically active. There was also low proportion of male participants with abdominal obesity (9.1%) in contrast to that observed for the females (52.1%). There were statistically significant differences between the sexes in tobacco use, alcohol consumption and abdominal obesity (Table 2).

No significant dyslipidaemia was observed among the participants. There is no participant with high TC value, and 4.4% had borderline high TC. TC ranged from 2.45 to 5.38 mmol/L (mean, 3.66 ± 0.64 mmol/L). In males, TC ranged between 2.58 and 5.38 mmol/L (mean, 3.54 ± 0.68 mmol/L), and in females, TC ranged between 2.45 and 5.32 mmol/L (mean, 3.73 ± 0.66 mmol/L). One female participant had borderline high LDL-C, and no high LDL-C level was recorded. LDL-C for all the participants ranged between 0.9 and 3.7 mmol/L (mean, 2.05 ± 0.58 mmol/L). In males, LDL-C ranged between 0.9 and 22.0 mmol/L (mean, 1.92 ± 0.52 mmol/L), and in females, LDL-C ranged between 0.95 and 3.7 mmol/L (mean, 2.12 ± 0.60 mmol/L). No participant recorded a high TG value, and less than 13% had a borderline high TG level (Table 3). Low HDL-C occurred in 30% of males and females.

**Table 3:** Lipid panel distribution according to sex

**Variables**	**Male (N = 30)**	**Female (N = 60)**	**Total (N = 90)**	**χ**^**2**^	**P-Value**
	**N (%)**	**N (%)**	**N (%)**		
**TC (mmol/L)**					
≤4.98	29 (96.7)	57 (95.0)	86 (95.5)	63.375	0.167
5.00–5.98	1 (3.3)	3 (5.0)	4 (4.5)		
≥6.00	0 (0.0)	0 (0.0)	0 (0.0)		
**LDL–C (mmol/L)**					
<2.50	27 (90.0)	42 (70.0)	69 (76.6)	63.750	0.251
2.50–3.23	3 (10.0)	17 (28.3)	20 (22.3)		
3.25–3.98	0 (0.0)	1 (1.7)	1 (1.1)		
≥4.00	0 (0.0)	0 (0.0)	0 (0.0)		
**TG (mmol/L)**					
<3.75	27 (90.0)	52 (86.0)	79 (87.5)	60.154	0.263
3.75–4.98	3 (10.0)	8 (14.0)	11 (12.5)		
≥5.00	0 (0.0)	0 (0.0)	0 (0)		
**HDL–C (mmol/L)**					
≤ 0.98	9 (30.0)	18 (30)	27 (30.0)	40.886	0.867
1.00 – 1.48	12 (40.0)	25 (41.7)	37 (41.2)		
≥ 1.50	9 (30.0)	17 (28.3)	26 (28.8)		
**Incomplete result**	3 (9.1)	11 (15.5)	14 (13.5)		

*TC* Total cholesterol, *LDL-C* Low density lipoprotein cholesterol, *TG* Triglyceride, *HDL-C* High density lipoprotein cholesterol.

## Discussion and conclusions

This study confirmed the hypothesis that most traditional risk factors for CVD are of low prevalence in rural dwellers of Ekiti in Ekiti State, Nigeria. Similar reports in other rural areas have been documented [21-24]. However, the prevalence of CVD risk factors has been shown to be on the increase both in Western and African countries, particularly in urban areas, while rural areas that have suffered from rural-urban migration are also becoming affected [25-27]. Community-based studies in rural areas in developing countries are lacking because of a lack of funding.

The participants in this study were predominantly in the age group 60–79 years (56.7%) and there was a preponderance of females, possibly because many of the young adults, particularly males, had migrated to the cities for work, and more males than females were engaged in crude farming activities, which have long been recognized as a risk factor for earlier death in men [28-30]. The very old formed the lowest proportion (17.3%) of the participants, which reflects the reduced life expectancy in low to middle income countries to which this community belonged [28]. Marital status, in which no males were widowed compared with 52.9% of females, may reflect the traditional practice in this community allowing men not only to marry more than one wife but to marry another wife in the event of the death of his wife. Furthermore, many young/middle-aged women were married to elderly men in the community. The majority of the participants belonged to the lower socioeconomic class, which was a reflection of their educational level (54.8% had no formal education) and therefore their source of income was predominantly peasant farming and petty trading. Besides this form of self-employed low-income work, the community did not have access to industrial or government employment.

Of all the modifiable risk factors evaluated, hypertension was found to be the highest burden for the participants. This is similar to earlier reports from rural communities in Ghana and Nigeria [31,32]; however, a higher prevalence of hypertension was demonstrated in this study. Combined systolic and diastolic hypertension (42.3%) was the commonest, followed by isolated systolic hypertension (ISH) (21.2%), and last, pure diastolic hypertension (2.9%). This may be explained by both the age structure of our population and the cut-off used to define hypertension. Over time, the cut-off value for hypertension has been lowered from 160/95 mmHg to 140/90 mmHg, which is bound to identify more people as having hypertension. As observed in this study and others from similar rural and semi-urban communities, there was an age-related increase in the prevalence of hypertension [31-33]. About 74% of our participants were above 60 years of age and therefore could be classified as elderly people, hence the possibility of a greater burden of hypertension. Significant numbers of the women were also past the menopause, which could also contribute to the burden of hypertension [34].

The overall prevalence (66.4%) of hypertension in this study was higher than in a study by Onwubere *et al*. [35] in Southeast Nigeria (46.4%). However, this study considered a population of 40–88 years of age while the study of Onwubere *et al*. included a population with an age range of 40–70 years and in a different Nigerian tribe. These factors may contribute to the difference as genetic factors are very important in the development of hypertension. The same reasons are also applicable to the prevalence of hypertension in a rural community in the Northern part of Nigeria (15.2%) reported by Okesina *et al*. [21]. The higher prevalence of ISH compared with pure diastolic hypertension in this study is expected, as the predominant age group was elderly and ISH is known to increase with age, unlike pure diastolic hypertension [36,37]. Combined systolic and diastolic hypertension as well as pure diastolic hypertension was more prevalent in males than females but the difference was not significant. The sex difference was similar to previous findings [7,36,37]. The prevalence of ISH was similar in both sexes.

The prevalence of obesity was 14.4%, with a higher rate in females (18.3%) than in males (6.0%). This may be related to the higher level of physical activity of men arising from their farming activities, while females engage in more sedentary activities such as trading. This is in accord with several previous studies [21,22,35]. Under-nutrition was also observed with a prevalence of 5.8%, and was commoner in males (6.1%) than in females (5.6%), though this was not a significant difference. This under-nutrition coexisting with obesity demonstrates a double burden in this community.

Abdominal obesity, as measured by waist circumference and perhaps a more sensitive measure of obesity in African populations [38,39], was observed in 38.46% of the participants, with a significantly higher rate in females (52.11%) compared with males (9.09%). This can be explained as a result of high caloric expenditure physical activities (farming) that predominate in males in this study compared with low caloric expenditure sedentary activities (trading) that predominate in females. This finding is similar to reports by Andre *et al.*[24] and Adegoke *et al*[40]. The smoking frequency among the participants was very low (2.9%), probably because of the religious nature of the community as well as poverty. Only a few of those who consumed alcohol (30.8%) were at high risk (1%), unlike the finding by Joel *et al.* in an African population [23], and this was also probably related to religion and poverty.

The low prevalence of diabetes observed in this study is similar to previous studies [35,36]. This may result from the absence of an affluent lifestyle and fewer problems with obesity when compared with more developed societies. The female preponderance was in tandem with other indicators of insulin resistance in this study, such as abdominal obesity (a well-known risk factor for metabolic syndrome and type II diabetes) [41,42], generalised obesity, and physical inactivity.

The high overall prevalence of overweight (29.8%)/generalised obesity (14.4%), truncal obesity (38.5%) and physical inactivity (29.8%) could partly be explained by a lack of access to leisure activity (recreation) in this community as well as a substantial proportion (26.9%) of the population in sedentary work (trading).

The lipid profile in this population was predominantly within the desired range, similar to previous reports [43,44], with almost no abnormalities in TC, LDL-C, and TG. The occupation of these individuals who are mainly farmers with high caloric expenditure may explain these results. Despite these observations, 30% of the participants had low HDL-C, which may not be unconnected with lack of dynamic (aerobic) exercise in this population as this probably is the most important among lifestyle interventions for raising HDL-C [45,46], and the similarities between males and females may be explained by the high number of postmenopausal females in whom the influence of oestrogen would be attenuated [46].

In conclusion, except for hypertension, obesity, physical inactivity and low HDL-C, the low proportion of most of the traditional cardiovascular risk factors such as smoking, high-risk alcohol consumption, diabetes, and high levels of TC, LDL-C, and TG was impressive. However, it is important to further emphasize the health benefits of reducing risk factors in an educational programme in this kind of community. The high prevalence of hypertension in this community is of concern, with possible sequelae of stroke, cardiac arrest, and heart failure. Therefore, this report highlights an urgent need for preventive and control educational programmes in a community of this nature.

## Competing interests

The authors declared that they have no competing interests.

## Authors’ contributions

OJO (corresponding author) designed the study, contributed to data collection and statistical analysis, wrote the protocol, and was involved in the first through to the final draft of the manuscript. OOA contributed to data collection and literature searches. OOO and BOA contributed to writing the manuscript. All authors have read and approved the final manuscript.

## Pre-publication history

The pre-publication history for this paper can be accessed here:

http://www.biomedcentral.com/1471-2261/13/89/prepub
